# Evaluation of Rheological Properties and Swelling Behaviour of Sonicated Scleroglucan Samples 

**DOI:** 10.3390/molecules17032283

**Published:** 2012-02-24

**Authors:** Siddique Akber Ansari, Pietro Matricardi, Chiara Di Meo, Franco Alhaique, Tommasina Coviello

**Affiliations:** Department of Drug Chemistry and Technologies, University “La Sapienza”, 00185 Rome, Italy; Email: siddiqueakber.ansari@uniroma1.it (S.A.A.); pietro.matricardi@uniroma1.it (P.M.); Chiara.Dimeo@uniroma1.it (C.D.M); franco.alhaique@uniroma1.it (F.A.)

**Keywords:** scleroglucan, sonication, rheology, polymer gels, swelling

## Abstract

Scleroglucan is a natural polysaccharide that has been proposed for various applications. However there is no investigation on its property variations when the molecular weight of this polymer is reduced. Scleroglucan was sonicated at two different polymer concentrations for different periods of time and the effect of sonication was investigated with respect to molecular weight variations and rheological properties. Molar mass, estimated by viscometric measurements, was drastically reduced already after a sonication for a few min. Sonicated samples were used for the preparation of gels in the presence of borate ions. The effect of borax on the new samples was investigated by recording the mechanical spectra and the flow curves. A comparison with the system prepared with the dialysed polymer was also carried out. The anisotropic elongation, observed with tablets of scleroglucan and borax, was remarkably reduced when the sonicated samples were used for the preparation of the gels.

## 1. Introduction

Scleroglucan (Sclg) is a homopolysaccharide, produced by fungi of the genus *Sclerotium*, consisting of a main chain of (1→3)-linked β-D-glucopyranosyl units bearing, every three units, a single β-D-glucopyranosyl unit linked (1→6). The commercial polymer is produced by various companies (e.g., Cargill, Mero-Rousselot-Satia, Degussa, CarboMer) with slightly differences in the application features.

Because of its peculiar rheological properties, its resistance to hydrolysis, temperature and electrolytes, Sclg has several industrial applications, in the oil industry, for water colours, printing inks, animal feed, *etc*. Several Japanese patents proposed the use of Sclg for the improvement of food quality. The polymer has been used also in cosmetic industry as well as in the field of pharmaceutics [1,2,3]. In the last few years, several studies have been carried out for the possible use of Sclg and its derivatives as new matrices for sustained drug delivery purposes [4,5,6,7,8,9,10,11,12]. Furthermore, Sclg shows antitumor, antiviral and antimicrobial activities as well as immune stimulatory effects [13,14,15,16].

X-ray diffraction studies indicate that Sclg has a triple-helical backbone conformation in the solid state that is maintained also in aqueous solutions [17]. In the presence of borax Sclg, like other polysaccharides, gives a weak gel that shows a higher capability to keep its shape when a stress is applied [18]. It is interesting to point out that when tablets were prepared with this Sclg/borax network, swelling induced a peculiar anisotropic elongation and, at the same time, also an uncommon enhanced diffusion of water molecules was found by means of NMR studies [19,20,21,22,23,24].

The Sclg/borax gel can be described in terms of soft nanochannels made up by Sclg triple helix aggregates. The triple helices are organized in domains with an intrinsic ordered structure that leads to an anisotropic arrangement of the chains along the compression direction during the tablet preparation. This kind of arrangement deeply influences the diffusion properties of water molecules (during the swelling experiments), which show an enhanced diffusion that may play an important role on the release performance of the gels. Actually, a deeper knowledge of the mechanisms involved in drug release also depends on matrix swelling and water molecules diffusion. 

The commercially available Sclgs have molecular weights of the order of 10^6^. This high molecular weight often is not suitable for specific applications, e.g., for the preparation of micro- or nano-systems suitable for drug delivery. In order to get a more versatile polymer, the decrease of the molar mass was suggested.

In this paper the preparation of various batches of Sclg (provided by Cargill) with reduced molecular weight, obtained by sonication, is described. The molecular weight was estimated by capillary viscometric measurements. The rheological properties of the new samples were investigated and a comparison with Sclgs purified by means of dialysis or centrifugation was carried out. Furthermore, the swelling behaviour of tablets prepared with Sclg obtained from different companies (samples already used for previous studies by the authors and, in some cases, no longer on the market or produced by companies acquired by other industries) and with different trade names and borax, together with tablets prepared with the sonicated samples, was studied in order to test the possible differences in water uptake and in anisotropic elongation among all tested samples.

## 2. Results and Discussion

### 2.1. Rheological Measurements

**Figure 1:**
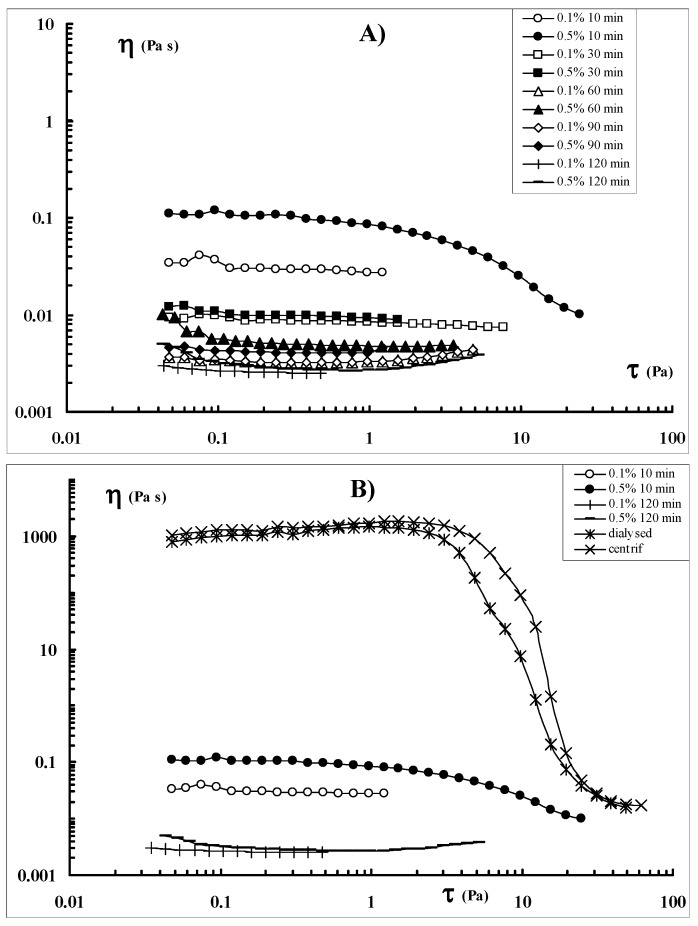
(A) Flow curves of Sclg samples sonicated at cp = 0.1% (open symbols) and cp = 0.5% (full symbols) for different periods of time. (B) Flow curves of Sclg sonicated for 10 and 120 min at cp = 0.1 and 0.5%. For comparison, also the flow curves recorded for the dialysed Sclg and the centrifuged Sclg, are reported (cp = 0.7%, T = 25 °C).

Sclg solutions were first prepared at two different polymer concentrations, cp = 0.1 and 0.5 (w/v), in order to test the influence of this parameter on the sonication process. Furthermore, the sonication process was carried out for different periods of time: from a minimum of 5 min up to 120 min. After sonication, the samples were centrifuged. The flow curves for the different batches of polymer sonicated for different periods of time at two different cp (cp = 0.1 and 0.5%) are shown in Figure 1. 

As a comparison, also the flow curves of the Sclg purified by dialysis, and the centrifuged Sclg, are reported. For the rheological measurements, all samples, regardless of the concentration used for the sonication process, were tested at cp = 0.7%. It can be noted that the Sclg dialysed and the centrifuged one, show similar flow curve profiles. It can be observed that a sonication of 30 min is sufficient to reduce drastically the viscosity of the polymer: at 𝜏 = 5 × 10^−2^ (Pa) the viscosity drops from 1,000 to 0.01 (Pa s) for both systems, (*i.e.*, the polymer that was sonicated at cp = 0.1% and the one that was sonicated at cp = 0.5%) indicating that polymer concentration during the sonication process is not a crucial parameter. A slight difference between the two set of cp could be appreciated only after a sonication of 10 min, being the viscosity of the polymer sonicated at cp = 0.5% slightly higher than that of the polymer sonicated at cp = 0.1%. Anyhow, ten min of sonication were sufficient to break significantly the Sclg chain backbones and to reduce to one half the molecular weight (see Section 3.2). 

**Figure 2:**
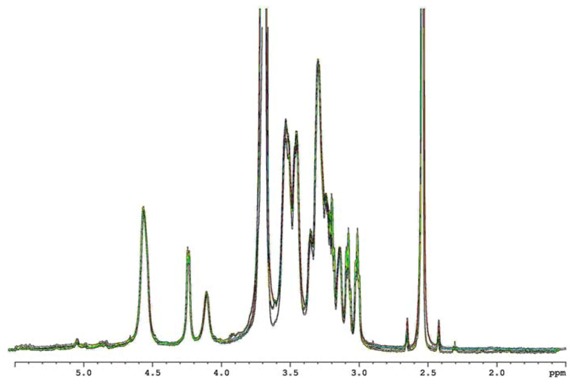
^1^H-NMR spectra at 600.13 MHz and 27 °C (DMSO-*d_6 _*+ 10% v/v of D_2_O) of dialysed Sclg (green), Sclg sonicated 30 min (red) and Sclg sonicated 60 min (black).

Increasing the sonication time, a further breakdown of the polymeric chains occurs without damaging the repeating unit structure, as evidenced by NMR analysis (Figure 2).

NMR was carried out in DMSO-*d_6_*, in order to separate the Sclg triple helices in single chains, thus obtaining sharp signals of the polymer; the addition of 10% v/v D_2_O allowed a further spectrum simplification by suppressing all hydroxyl proton resonances. As shown in Figure 2, the spectra of the dialysed sample, of the Sclgs sonicated for 30 and 60 min, are superimposable. In particular, the ratio between the anomeric β (1→3) protons at 4.571 ppm and the anomeric β (1→6) protons at 4.245 ppm remains constant and equal to 3 for all tested samples. Thus, the sonication process can be proposed as a valid method for the Sclg molecular weight reduction. In fact, by means of sonication, it is possible to prepare Sclg with a wide range of molecular weights but keeping intact the molecular structure as supported by the experimental conditions that prevent disentanglements of the triple helices: temperature below 135 °C (melting temperature of the triple helix), because of the ice bath; environmental pH well below 13 and absence of DMSO (solvent capable to breakdown the Sclg triple helix). This is a crucial point, especially with reference to possible industrial and biomedical applications of Sclg, since the rheological properties of the polymeric solutions prepared with the sonicated samples show remarkable variations in comparison to the dialysed polymer.

**Figure 3:**
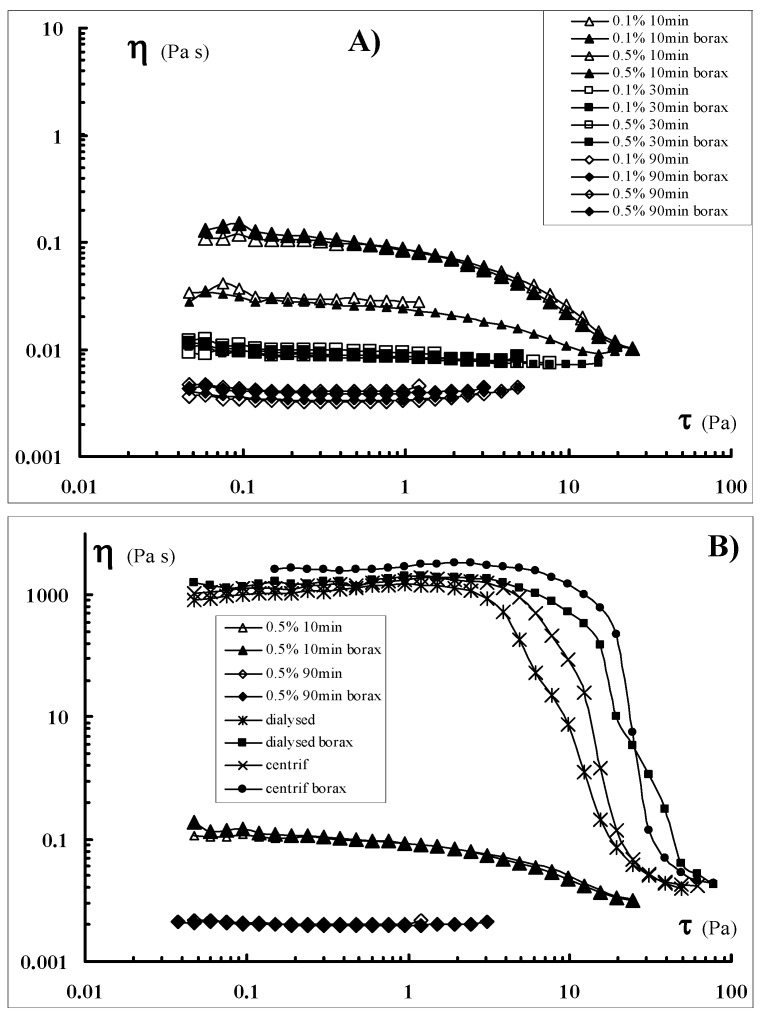
Flow curves of Sclg samples (cp = 0.7%), previously sonicated at cp = 0.1% and cp = 0.5%, with (full symbols) and without borax (empty symbols) (A). Flow curves of Sclg sonicated (cp = 0.5%) for 10 and 90 min with and without borax: for comparison, also the flow curves recorded for the dialysed and the centrifuged Sclgs, are reported (B).

The addition of borax to the various samples was also investigated (Figure 3). Addition of borate ions to Sclg samples led to the formation of a network with a higher flow resistance (in comparison to the polymer alone) that was also capable to keep its shape (*i.e.*, a self-sustaining gel is formed). 

Critical stresses, 𝜏_c_ = 2 and 𝜏_c_ = 4 were found for Sclg and Sclg/borax respectively (using the dialysed Sclg) and 𝜏_c_ = 3 and 𝜏_c_ = 6 were found for Sclg and Sclg/borax (using the centrifuged Sclg), where 𝜏_c_ represents the stress value corresponding to 95% of the viscosity plateau value. On the other side, for all sonicated samples, no appreciable variations were detected in the flow curves, regardless of the presence or absence of the borate ions. Thus, in these samples, the key parameter for the flow characteristics is only represented by the molecular weight of the polymer.

**Figure 4:**
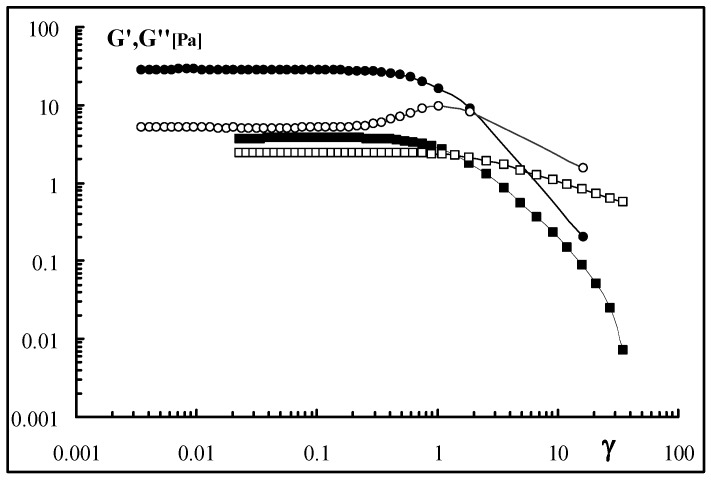
Viscoelasticity plot for dialysed Sclg (●, ○) and for Sclg sonicated for 5 min (■, □); cp = 0.7%, T = 25 °C (G’ = full symbols; G” = empty symbols).

Interestingly, the modulus variations in the stress sweep experiments also show the noticeable effect of the sonication process on the supramolecular structure. Actually, in order to acquire meaningful data for the sonicated samples, a sonication of only 5 min was applied (Figure 4). In fact, for higher sonication times, it was not possible to detect the sample moduli. The effect of 5 min sonication on the storage modulus is rather impressive: for G’: a drastic reduction of ten times was observed while the decrease in the loss modulus, G”, was much smaller. Thus, the breakdown of the polymeric chains mainly influences the elastic component of the system, *i.e*., the chains, becoming shorter and shorter, are not capable any longer to give entanglements with effective elastically crosslinking points which are responsible for the increase of G’ in the gel system. On the other side, also the viscous component is influenced by the molecular weight decrease with a corresponding lowering (even smaller) of G” that is comparable and almost the same of G’.

**Figure 5:**
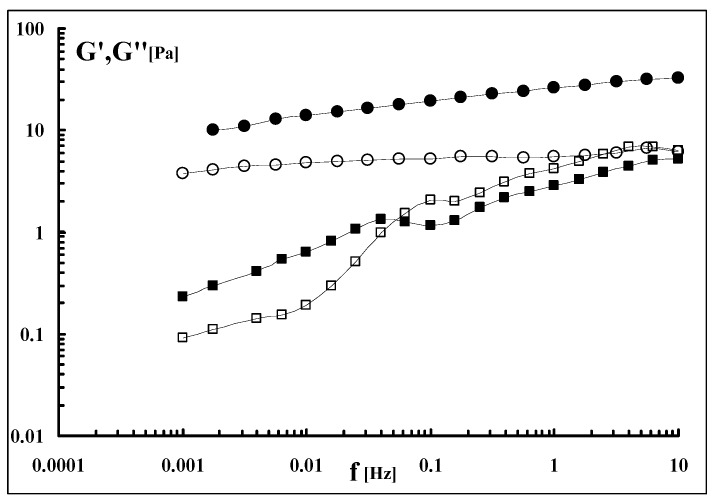
Mechanical spectra of dialysed Sclg (●, ○) and of Sclg sonicated for 5 min (■, □); cp = 0.7%, T = 25 °C (G’ = full symbols; G” = empty symbols).

Interesting was also the comparison between the two mechanical spectra (Figure 5). While the dialysed Sclg showed the typical weak gel behaviour, the sample sonicated for 5 min showed the characteristic sol-gel transition. This means that a significant reduction in the molecular weight (see also the viscosity measurements section) dramatically influences also the mechanical properties of the sample. 

For frequencies lower than 0.05 the system is liquid-like while for higher frequencies the system is solid-like. A sonication prolonged for 10 min or higher leads to liquid-like systems with moduli too low to be experimentally recorded.

### 2.2. Viscosity Measurements

**Figure 6:**
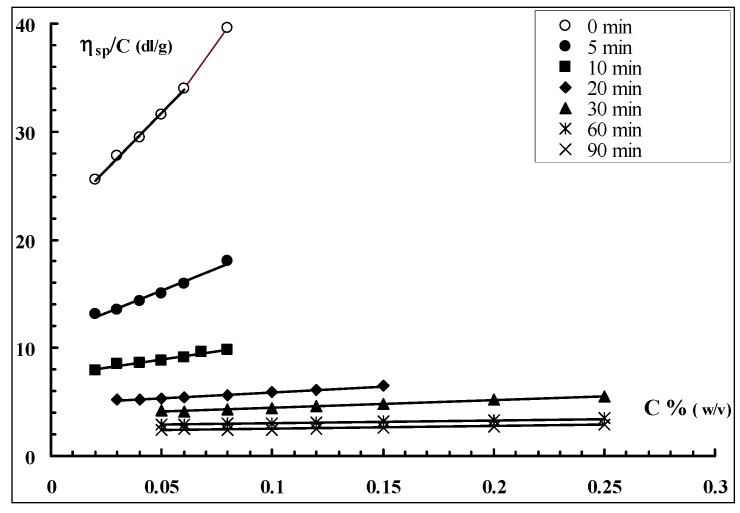
Plot of η_specific_/c *vs.* polymer concentration for Sclg samples sonicated for different periods of time (NaOH = 0.01 N, T = 25 °C).

Solutions of the sonicated polymer were prepared in 0.01 N NaOH in order to break the possible aggregates still present in solution [25,26]. Viscosity measurements were carried out at 25 °C and the results are shown in Figure 6. 

From the intercepts, *i.e.*, from the intrinsic viscosities [η] of the samples, according to the Mark-Houwink-Sakurada equations valid for Sclg [27]:

[η] = KM_w_^1.7^ for M_w _< 5 × 10^5^

**Figure 7:**
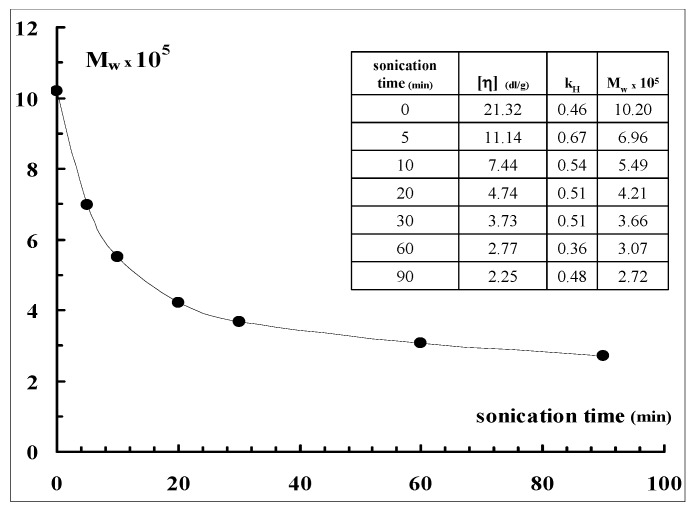
Sclg molecular weight dependence on sonication time (NaOH = 0.01 N, T = 25 °C), together with the calculated Huggins constants (k_H_).

[η] = KM_w_^1.2^ for M_w _> 5 × 10^5^

where K = 1,3 × 10^−7^ cm^3^ g^−1^, the molecular weights of the various fractions were estimated and reported in Figure 7. It is possible to observe that, applying the sonication for only 10 min, the molecular weight of the polymer was reduced to one half. The reduction was further increased by increasing the sonication process for longer time. The values of the Huggins constant (see Table in Figure 7) calculated for all samples from the slope of the linear trend of viscosity data are 0.5 ± 0.1 indicating that, in the applied experimental conditions (0.01 N NaOH), no significant aggregation of the polymeric chains occurs. 

### 2.3. Water Uptake and Dimensional Increase Studies

It is known [19,20,21,22,23,24] that tablets, prepared with the Sclg/borax freeze-dried hydrogel, undergo an anomalous peculiar swelling essentially along one direction. New tablets were then prepared with the sonicated samples and the water uptake and the elongation behaviour were followed as a function of time. As a comparison, the experiments were carried out also on tablets prepared with Sclg produced (in some cases not marketed anymore) by different companies, *i.e.*, Degussa, CarboMer, and Mero- Rousselot-Satia (Actigum CS11).

**Figure 8:**
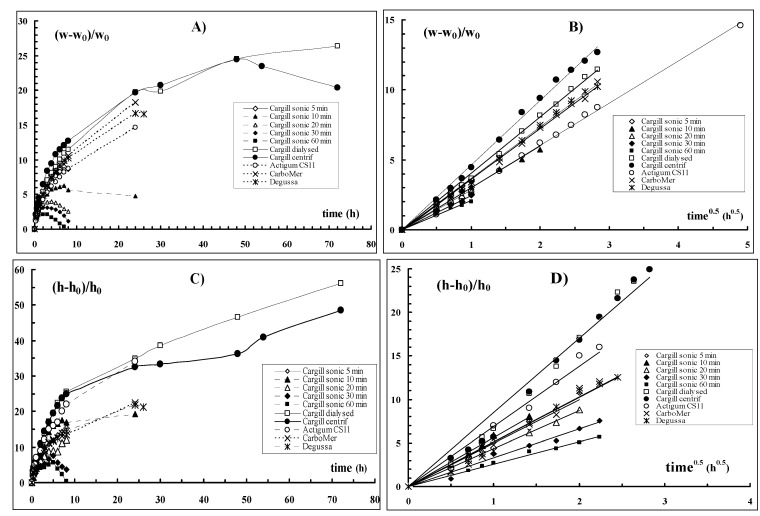
Water uptake ((w-w_0_)/w_0_; (A) and relative height increase ((h-h_0_)/h_0_; (C) from tablets of Sclg/borax prepared with Sclg of different brands (Cargill dialysed, Cargill centrifuged, Actigum CS11, CarboMer and Degussa) and with Sclg sonicated for different periods of time (Cargill sonicated for 5, 10, 20, 30 and 60 min). (A) and (C): as a function of time; (B) and (D): as a function of the square root of time. Experiments were carried out in triplicate and the obtained values always laid within 10% of the mean.

The results, reported in Figure 8 (in distilled water at 37 °C), clearly show that both, the water uptake and the anisotropic elongation of the tablets, observed for the Sclg/borax system, were dramatically reduced when the sonicated samples were tested. 

While the tablets prepared with the untreated polymer increased their weight and elongated even after 3 days, those prepared with Sclg sonicated for 5 min started to dissolve in the medium already after 8 hours. This phenomenon occurred earlier and earlier as the applied sonication time increased: the tablets prepared with Sclg sonicated for 1 hour (M_w _ = 3.07 × 10^5^) started to loose their weight already after 1 hour and the anomalous elongation could be detected only up to 5 hours. After 8 hours the tablets dissolved completely in the swelling medium.

**Figure 9:**
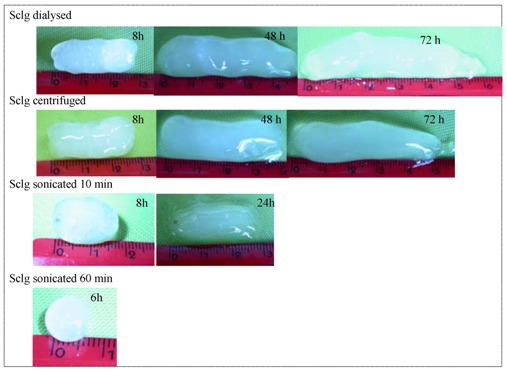
Pictures of Sclg/borax tablets, prepared with Cargill Sclg dialysed, centrifuged and sonicated for 10 and 60 min, after swelling for different periods of time in distilled water at 37 °C (the height of the tablets before the swelling process was h_0_ ≈ 1.05 mm).

In Figure 9 the pictures of tablets prepared with different types of Sclg and swelled for different periods of time are shown. Both processes, the uptake of water and the height increase, obey, during the first hours, the fickian law in all tested systems (Figure 8). Thus, the reduction of the polymeric chain lengths did not change the diffusional character of water molecules behaviour during the imbibition of the tablet matrices.

**Figure 10:**
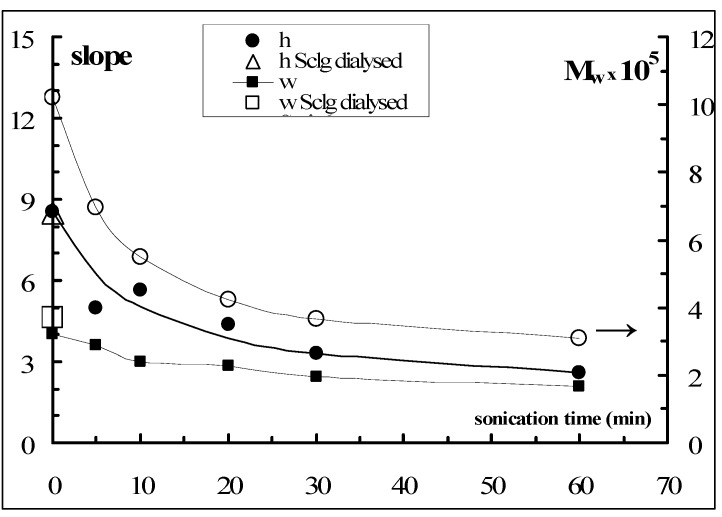
Slopes of the fickian linear trends for the water uptake and height increase (left) together with the molecular weight dependence on the sonication time (right).

In Figure 10 the rates of water uptake and tablet elongation processes (*i.e.*, the slope of Figure 8 linear trends) *vs.* the sonication time are reported. For comparison, the dependence of M_w_ on sonication time is also shown. It is clear that the highest rate in anisotropic elongation is observed for tablets prepared with Sclg with M_w_ = 6.96 × 10^5^. Also in the profile describing the M_w_ *vs.* the sonication time the biggest effect is observed after a sonication of 5 min. On the other hand, the weight increase showed a slower kinetic in comparison to the height increase and a reduced dependence on the sonication time. As a comparison the rates calculated for the swelling of tablets prepared with the dialysed Sclg are also reported. Both rates are almost superimposable to those of centrifuged Sclg. Thus, the water uptake and anisotropic elongation phenomena mainly depend on the polymeric molar mass, both for the extent of the processes and for the rate. On the other side for the raw polymer, the dialysis purification (in order to eliminate the low mass fraction of the polymer and impurities, if present, of low molecular weight) or the centrifugation process (to eliminate the large and rough aggregates) did not influence significantly the swelling behaviour.

## 3. Experimental

### 3.1. Materials

Sclg was provided by Cargill (Minneapolis, USA), while borax and NaOH Normex were Carlo Erba (Italy) products. For the sample preparations distilled water was always used, except for NMR experiments, where deuterium oxide (D_2_O) (Cambridge Isotope Laboratories, USA) was used. All products and reagents were of analytical grade. 

For an appropriate comparison, also scleroglucans provided in the past by different companies, were tested: in particular the Sclg provided by Mero-Rousselot-Satia (France, molecular weight = 1.4 × 10^6^ from viscometric measurements), named Actigum CS11 (no more in the market), the Sclg provided by CarboMer (USA, degree of polymerization = 800), and the Sclg provided by Degussa (Germany, molecular weight = 1.1 × 10^6^, from viscometric measurements).

#### 3.1.1. Purification of Scleroglucan

A given amount of polymer was dissolved in distilled water (polymer concentration, cp = 0.5% w/v), and then kept under magnetic and mechanical stirring at room temperature for 24 h. The obtained solution was exhaustively dialysed at 7 °C against distilled water and then freeze-dried.

#### 3.1.2. Hydrogel and Tablet Preparation

For the preparation of the tablets, an appropriate amount of polymer (about 200 mg) was magnetically stirred in water for 24 h. Then, the calculated amount (*i.e.*, moles of borax = moles of repeating units of polymer) of 0.1 M borax solution was added and the system was left under magnetic stirring for 5 min The obtained sample (cp = 0.7%, w/v) was kept overnight at 7 °C for gel-setting and then freeze-dried. Tablets were prepared from the freeze-dried sample with an IR die (Perkin Elmer hydraulic press) using a force of 5.0 kN for 30 s. The weight of the Sclg/borax tablets was 230 ± 10 mg, the diameter was 13.0 ± 0.1 mm, and the thickness was 1.05 ± 0.02 mm. 

### 3.2. Methods

#### 3.2.1. Sonication

To reduce the Sclg molecular weight a High Intensity Ultrasonic Processor (750 Watt model, probe type sonicator–Vibra Cell–VC 750, Cole-Parmer, USA) was used, working at 20 kHz, with a 6.5 mm microtip, applying an amplitude of 30% and pulser cycles of 30 s ON and 30 s OFF. Aqueous solutions (50 mL) of Sclg, prepared at two different polymer concentrations, cp = 0.1 and 0.5% w/v, were kept in an ice bath during sonication. All samples (cp = 0.1 and 0.5% w/v), after sonication, were centrifuged for 20 min at 18,000 rpm and 20 °C in order to remove the probe-tip residues from the polymer solutions. Freeze-drying procedure was then carried out.

#### 3.2.2. Ultracentrifugation

Sclg samples were also prepared by centrifugation, in order to separate the aggregates from the polymer solutions (cp = 0.5% w/v). For this purpose a centrifuge Sorvall WX 80 ULTRA (Thermo Scientific, USA) was used (20 min at 18,000 rpm and 20 °C). Freeze-drying procedure was then carried out.

#### 3.2.3. NMR Analysis

Samples of dialysed Sclg, Sclg sonicated 30 min and Sclg sonicated 60 min, about 4 mg, were solubilized in 630 μL of DMSO-*d_6_* + 70 μL D_2_O. ^1^H-NMR experiments were performed at 27 °C on a Bruker AVANCE AQS 600 spectrometer operating at 600.13 MHz and equipped with a Bruker multinuclear, *z*-gradient probe head. A soft presaturation of the HOD residual signal was applied before the spectra acquisition [28].

#### 3.2.4. Rheological Measurements

The rheological characterization of the Sclg and Sclg/borax samples was performed by means of a controlled stress rheometer (Haake Rheo-Stress RS300; Thermo Haake DC50 water bath); a cross-hatch plate device (Haake PP35 TI: diameter = 35 mm) was used in order to reduce the extent of wall slippage phenomena [29]. To perform the measurements, the samples were transferred in the rheometer and the upper plate was then lowered until it reached the sample surface. Gap-setting optimizations have been undertaken according to the procedure described elsewhere [30]. Samples were loaded at fixed temperature (25 °C), and coated around their periphery with light silicone oil to minimize loss of water. Rheological properties were studied under small and large deformations, as well as in flow conditions, by applying different procedures: stress (ν **=** 1 Hz, ω = 2πν = 6*.*28 rad/s) and frequency sweep (in the linear viscoelastic region; constant deformation γ = 0*.*01).

#### 3.2.5. Viscosity Measurements

For the viscosity measurements an automatic viscometer (Instrument Schott AVS 370, Lauda, Germany) with a water bath (Lauda 0.15 T) allowing the temperature control to 0.1 °C was used. An Ubbelohde capillary viscometer (Type No 531 01, with a capillary diameter = 0.54 mm, Schott-Geräte) for dilution sequences, with a flux time for the solvent (NaOH = 0.01 N) at 25 °C of 178.56 s, was used.

The polymer solutions (Sclg in 0.01 N NaOH), before measurements, were filtered two times with 1.2 μ Millipore filters. The solvent for the dilution was previously filtered three times with 0.22 μ Millipore filters. The flux time of the solution (η) was compared with that of the solvent (η_0_) and the relative increase of fluxing time (η/η_0_ =η_rel_; η_rel_ − 1 = t/t_0_−1 =η_specific_) was evaluated at different polymer concentrations. In the range of viscosities up to about twice that of water (*i.e.*, η_rel_ = 2) the following Huggins equation is valid:

η_specific_/c = [η] + *k*_H_[η]^2^c

The limiting value of η_specific_/c for c →0 represents the intrinsic viscosity, [η] (cm^3^/g), while from the slope of the linear trend of viscosity data it is possible to calculate the Huggins constant, which gives an indication on the aggregation state of the polymer in the tested conditions of solvent and temperature [31]. The intrinsic viscosity is an important parameter being related, according to the Mark-Houwink-Sakurada equation, to the molar mass of the sample [32]: [η] = *K*M_w_*^a^*, where *K* and *a* are constants for each polymer-solvent system at a given temperature.

#### 3.2.6. Water Uptake and Dimensional Increase Studies

The swelling of Sclg/borax tablets, prepared with different kinds of Sclg (*i.e.*, Cargill dialysed, Cargill centrifuged, Cargill sonicated for different times, Actigum CS11, CarboMer and Degussa), was carried out by soaking the tablets in distilled water at 37 °C. At fixed time intervals, the tablets were withdrawn, the excess of water was removed with soft filter paper for 5 s, and then the corresponding weights and dimensional variations along the longitudinal axis were determined by means of a screw gauge with an accuracy of ± 0.1 mm. No remarkable variations of cross-section dimensions were detected during the swelling process. All experiments were carried out in triplicate and the obtained values always laid within 10% of the mean.

## 4. Conclusions

The sonication process did not change the basic repeating units of Sclg, as evidenced by means of NMR spectra. Thus, sonication appeared a rather easy and suitable method to reduce the molecular weight of Sclg without destroying the structural characteristic of the polymeric chains. The effect of the M_w_ reduction on the rheological properties was studied. The flow curves were noticeably modified as a consequence of the polymeric chain reduction. Furthermore, it was possible to acquire the mechanical spectra only for the sample sonicated 5 min, the moduli of the other samples being too low to be detected. The sample sonicated for 5 min lost the characteristic of a weak gel (typical of Sclg samples) and showed the peculiar spectra of a system undergoing the sol-gel transition. Also the addition of borax, that usually increases the flow resistance in Sclg samples, did not affect the sonicated samples. 

The Sclg molecular weight, as estimated by viscometric measurements, decreased very rapidly by applying ultrasounds to the polymeric solutions. The reduction to one half of M_w_ occurred already after 10 min of sonication.

The swelling behaviour of Sclg/borax tablets was also investigated. In this respect the reduced molecular weight of the polymer led to a noticeable difference in water uptake and anisotropic elongation in comparison to the dialysed Sclg. The tablets started to lose weight and to swell along one direction much earlier than the reference system showing the polymeric chain length as the most important parameter for these processes. 

It has to be underlined that the possibility to modulate the mechanical properties of the Sclg solutions by changing the polymer molecular weight is very important in order to rationalize its behaviour in formulations to be used in various fields, as in drug delivery, for biomedical and cosmetics applications, and in food industry.
